# Strengthening evaluation and implementation by specifying components of behaviour change interventions: a study protocol

**DOI:** 10.1186/1748-5908-6-10

**Published:** 2011-02-07

**Authors:** Susan Michie, Charles Abraham, Martin P Eccles, Jill J Francis, Wendy Hardeman, Marie Johnston

**Affiliations:** 1 Department of Clinical, Educational and Health Psychology, University College London, London, UK; 2 Peninsula College of Medicine and Dentistry, Exeter, UK; 3 Institute of Health & Society, Newcastle University, Newcastle, UK; 4 Health Services Research Unit, University of Aberdeen, Aberdeen, UK; 5 Department of Public Health and Primary Care, University of Cambridge, Cambridge, UK

## Abstract

**Background:**

The importance of behaviour change in improving health is illustrated by the increasing investment by funding bodies in the development and evaluation of complex interventions to change population, patient, and practitioner behaviours. The development of effective interventions is hampered by the absence of a nomenclature to specify and report their content. This limits the possibility of replicating effective interventions, synthesising evidence, and understanding the causal mechanisms underlying behaviour change. In contrast, biomedical interventions are precisely specified (*e.g.*, the pharmacological 'ingredients' of prescribed drugs, their dose and frequency of administration). For most complex interventions, the precise 'ingredients' are unknown; descriptions (*e.g.*, 'behavioural counseling') can mean different things to different researchers or implementers. The lack of a method for specifying complex interventions undermines the precision of evidence syntheses of effectiveness, posing a problem for secondary, as well as primary, research.

We aim to develop a reliable method of specifying intervention components ('techniques') aimed at changing behaviour.

**Methods/Design:**

The research will be conducted in three phases. The first phase will develop the nomenclature. We will refine a preliminary list of techniques and definitions. Using a formal consensus method, experts will then define the key attributes of each technique and how it relates to, and differs from, others. They will evaluate the techniques and their definitions until they achieve an agreed-upon list of clearly defined, nonredundant techniques. The second phase will test the nomenclature. Trained experts (primary researchers and systematic reviewers), equipped with a coding manual and guidance, will use the nomenclature to code published descriptions of complex interventions. Reliability between experts, over time, and across types of users will be assessed. We will assess whether using the nomenclature to write intervention descriptions enhances the clarity and replicability of interventions. The third phase will develop a web-based users' resource of clearly specified and nonredundant techniques, which will aid the scientific understanding of, and development of, effective complex interventions. Dissemination throughout the project will be through stakeholder meetings, targeted multidisciplinary workshops, conference presentation, journal publication, and publication in an interactive web-based platform (a Wiki).

**Discussion:**

The development of a reliable method of specifying intervention components aimed at changing behaviour will strengthen the scientific basis for developing, evaluating, and reporting complex interventions. It will improve the precision of evidence syntheses of effectiveness, thus enhancing secondary, as well as primary, research.

## Background

Behaviour change interventions (*e.g.*, to increase physical activity, adherence behaviours, screening attendance) are typically complex, involving many interacting components. We need methods of specifying and reporting complex interventions in order to strengthen the knowledge base required for such interventions to be more effective, replicable, and implementable. The complexity of interventions to change behaviour is determined, in part, by the number of components involved. Components include the techniques to facilitate behaviour change that constitute the active ingredients of the intervention and procedures for delivery of those techniques. Procedures for delivery include who delivers the intervention, to whom, how often, for how long, in what format, and in what context [1]. The UK Medical Research Council's guidance [2] for developing and evaluating complex interventions acknowledges the need for improved methods of specifying and reporting intervention content. The CONSORT statement for randomised trials of nonpharmacologic interventions calls for precise details of the intervention, including a description of the different intervention components [3]. It is important to specify and report both the active techniques and procedures for delivery. This protocol focuses on the former, that is, methods for specifying what is delivered, rather than on how it is delivered. It addresses techniques that target the behaviour of individuals but that may be delivered in a variety of ways (*e.g.*, prompts or reminders delivered by a 'buddy', telephone call from a healthcare professional, postal leaflet, or environmentally, such as hand-washing signs). By technique, we mean a replicable component of an intervention designed to alter or redirect causal processes that regulate behaviour; that is, a technique is proposed to be an 'active ingredient' (*e.g.*, feedback, self-monitoring, and reinforcement). Techniques also have specified criteria for their operationalisation, that is, minimum delivery specifications that would allow identification of that technique (*e.g.*, feedback must involve providing the target audience with information about their behaviour). The identification of behaviour change techniques (BCTs) is critical to understanding how organisational change and national policy changes (including policies around access and price) have their effects on individuals' health-related behaviours.

Despite the considerable investment in randomised controlled trials (RCTs) of complex interventions and in systematically reviewing their effects, interventions tend to be poorly described and reported. There is no consensus on terminology, and descriptions of interventions lack the specificity required for replication [2,4-6]. When secondary data analyses are conducted to ascertain which types of interventions are effective, many are too poorly specified to be included [7]; there is no consensus on how to classify content, and, therefore, each analysis develops its own classification system [1,8]. This results in much wasted effort since there is no common method for synthesising the findings of primary studies in a conceptually coherent way. Further, unless we can specify the active BCTs delivered within the standard care or control group, replication and accurate implementation is difficult or even impossible, and effect sizes for new interventions will continue to be uninterpretable [9]. These problems are evident across a wide variety of large, expensive trials of public health [10-12] and implementation interventions [13]. This impedes the accumulation of knowledge and implementation of effective behaviour change interventions [14]. In a 2008 address, the President of the Association for Psychological Science stated, 'For psychological research to flourish and develop into an increasingly cumulative basic science, there are some fundamental requirements. It's essential to develop and use common shared tools and a common language, so that replication, and building on solid work, becomes accepted practice and is valued (http://www.psychologicalscience.org/observer/getArticle.cfm?id=2430).'

Scientific advance requires an agreed-upon and reliable method of specifying and labeling BCTs [15]. Behavioural science has provided us with myriad potential BCTs [5,15,16], but there is no agreement on how they are labeled and identified. The same technique may be described by different labels (*e.g.*, 'self-monitoring' may be labeled 'daily diaries'), and the same labels may be applied to different BCTs (*e.g.*, 'behavioural counseling' may involve 'educating patients' or 'feedback, self-monitoring, and reinforcement' [17]). Imprecise labeling may lead to misleading conclusions in evidence synthesis. As a result of under-specification, behavioural medicine researchers and practitioners have been found to report low confidence in their ability to replicate highly effective interventions for diabetes prevention [5]. This problem needs to be solved to strengthen behavioural science and improve behaviour change intervention effectiveness.

Despite recommendations for describing intervention components [2,3], no rigorous and widely accepted methodology for doing this has been suggested. We propose to develop a systematic, referenced nomenclature (a system of technical terms used in a science, such as the periodic table of elements in chemistry or the biological classification) of BCTs with fully operationalised definitions to enable replication. This will form the basis of a future hierarchical classification (or 'taxonomy'). Describing behaviour change intervention content by a systematically produced, reliable nomenclature will strengthen the following:

1. Knowledge base: Published reports of intervention studies will be able to provide more detail on the BCTs, making effective interventions easier to replicate in primary research. They will also be able to specify 'standard care', thus ensuring that evaluated interventions are actually different from standard care comparator conditions. Systematic reviewers will be able to use a reliable method for extracting information about intervention content, thus identifying and synthesising discrete, replicable, potentially active ingredients associated with effectiveness. A shared language has allowed us and other reviewers to use an early version of a technique nomenclature to synthesise heterogeneous interventions and use meta-regression to determine which component BCTs are effective [8,9,15,18,19]. For example, a systematic review of 122 evaluations of interventions to increase physical activity and healthy eating [7] found that the technique 'self-monitoring' explained the greatest amount of among-study heterogeneity (13%). Interventions that combined self-monitoring with at least one other theoretically derived technique were significantly more effective than the other interventions (pooled effect sizes of 0.42 vs. 0.26, respectively). Another example of a study using this method reanalysed a Cochrane review of audit and feedback interventions and allowed the investigation of the separate effects of goal setting, monitoring, and action plans [20].

2. Evaluation and implementation: In RCTs evaluating behaviour change interventions, effect sizes will be interpretable in the context of clear specification of both intervention and control groups. Intervention developers will be able to use a comprehensive list of BCTs (rather than relying on the limited set they are aware of) to produce guidelines about how to operationalise the BCTs in protocols for implementation.

### Development of a preliminary list of BCTs

As a first stage, we have reliably identified a set of 26 BCTs from 195 published descriptions of behaviour change interventions to increase physical activity and healthy eating [15], demonstrating the feasibility of a method for developing standardised labels and definitions of BCTs included in complex interventions and specifying behaviour change interventions in terms of a defined list of BCTs. We subsequently extended this list to a wider range of behaviours, drawing on systematic reviews [21] and an analysis of relevant textbooks, reliably identifying 54 BCTs [5]. Further work is needed to extend the list to a wider range of types of behaviour and to improve the definitions of approximately 50 additional BCTs that were poorly specified (some similar BCTs were referred to by a variety of labels, and some labels were unclear or overlapping). More recently, we, and others, have extended the list to techniques designed to change other behaviours (*e.g.*, smoking) and populations (*e.g.*, obese patients) [18,19]. At this stage, we are limiting the project to behaviour change interventions targeting individual behaviours because of the time and resources required, while recognising the need to extend this to other types of complex interventions that target different levels of healthcare systems [22].

### Anticipating uptake

Few systematic reviews of behaviour change interventions use nomenclature systems [21,23,24]. Our preliminary BCT list, developed and evaluated using systematic methods to assess interrater reliability, has been widely used internationally, within a short period of its publication (2008), to report interventions [17], synthesise evidence [9,18,19], and design interventions [25]. Subsequently, we were invited to write journal editorials that have influenced editorial policy, requiring specification of complex intervention components to be based on reliable methods (*e.g.*, in *Addiction *and *Implementation Science*), and a group of 12 international journal editors have built on this to widen the call for developing reporting methods, forming the Workgroup for Intervention Development and Evaluation Research (WIDER, http://interventiondesign.co.uk/?page_id=9). This evidence of uptake supports the need for, and usability of, a nomenclature system.

Given the impact of our initial work, it is important to extend, consolidate, and enhance the generalisability of this method by building a wider, international consensus and disseminating and evaluating the nomenclature.

### Aims

Our goals for this project are as follows:

1. To develop a reliable and generalisable nomenclature of BCTs as a method for specifying, evaluating, and implementing complex behaviour change interventions

2. To lay a foundation for

a. a comprehensive methodology that can be applied to many different types of complex interventions, including organisational and community interventions

b. a fully developed, hierarchically organised taxonomy of BCTs

3. To achieve multidisciplinary and international acceptance and use to allow for its continuous development

### Objectives

Our objectives for this project are as follows:

1. Development: Generate an extensive list of clearly labeled, defined, nonredundant BCTs as the basis of the nomenclature (phase 1).

2. Evaluation: Test the reliability and usability of the preliminary nomenclature across different behaviours and populations (phase 2).

3. Prototype nomenclature: Produce a nomenclature with definitions and guidance on its use, evidence of consensus, evidence of reliability, and usability of each BCT, illustrated with examples from effective interventions (phase 3).

4. Implementation and dissemination: Make the nomenclature and its method of development widely accessible through a systematic dissemination plan (cross-phase).

## Methods

### Phase 1: addressing objective 1: consensus development

#### Phase 1a: recruiting experts and leaders (two months)

We have had an extremely positive response to our invitations to participate: 20 US, European, and UK multidisciplinary experts and six research centers, comprising about 30 experts, are motivated to work with us in developing the nomenclature. We have identified a further 20 leaders in the field of interventions; since we need a total of 74, we will also 'snowball' via our collaborators and research and professional networks. Experts include members of the US National Institutes of Health's Behaviour Change [26] and Health Maintenance Consortia (http://hmcrc.srph.tamhsc.edu/default.html; accessed 25.6.09).

#### Phase 1b: developing the nomenclature: clarifying and refining the list using a Delphi survey of expert users (eight months)

### Objective

Our objective for this phase was to refine and clarify the preliminary list of 54 BCTs [5] and develop the nomenclature.

### Participants

Participants will comprise expert users, including researchers who design and evaluate complex behaviour change interventions, and practitioners who apply the BCTs. Whilst for this method a (homogeneous) panel size of 12 optimises efficiency and reliability [27], we will allow a slightly larger group to ensure representation of a range of disciplinary perspectives. We will also allow for an increase in the number of people surveyed at subsequent stages of the survey as appropriate.

## Method

The Delphi method [28], a consensus development method that uses two-way, iterative information exchange, will be used. The study material will be prepared by the project researcher, who will agree with the investigators on initial working definitions for the 54 BCTs reliably extracted from textbooks. We will ask panelists to read the list, identify redundancy in the behaviour change techniques, clarify any remaining techniques that are unclear as far as possible by redefinition or adding or subtracting components, and identify any omitted BCTs. We will then present panelists with the refined list of techniques, with each technique having its key definitional attributes highlighted. In a formal questionnaire survey, we will ask them to rate (on a 1 to 9 scale) the following: (a) for each highlighted attribute within each technique, whether it is necessary; (b) whether there are any attributes that are missing, and if so, what they are; (c) for each overall technique as a whole, whether it is clear, precise, and distinct; (d) if their answers to (c) are ratings of 6 or lower, they will be asked whether their scores on (a) and (b) explain their low score on (c). This will allow us to amend technique definitions. Any technique that is rated by the panel as being appropriately defined, having no missing attributes, and being clear, precise, and distinct (as judged by ratings of 7 or more) will be judged to be defined. The remaining techniques will be elaborated on and/or refined in response to the first round of scoring and will then be sent to the panel again, with the same questions asked. BCTs that are still not viewed as being defined adequately after this second round will be examined to ascertain whether a further round is likely to generate a consensual definition. Techniques with scores from 4 to 9 may be subject to a further round. BCTs that attract a wide range of ratings (particularly in the 1 to 6 range), with no obvious agreement, will be regarded as indefinable. We estimate that this task will take each expert two hours for the first round and one hour for subsequent rounds.

### Analyses

The ratings will allow the key definitional characteristics of each technique to be identified. Group scores of the ratings for clarity, precision, and distinctiveness and indices of spread will be calculated. The investigators will complete further work on poorly understood or poorly defined BCTs to split them into component parts, relabel them, or reject them as indefinable.

### Product

The result of this analysis will be a refined list of distinct, clearly and precisely defined BCTs (*e.g.*, 'graded task' might be defined as '1. set easy tasks to perform; 2. set increasingly difficult tasks; 3. until target behaviour is performed'). On the basis of redundancy and overlap in preliminary searches, we anticipate that fewer than 90 BCTs will be defined through this process.

### Phase 2: addressing objective 2: evaluating the nomenclature

#### Phase 2a: nomenclature training resource materials (six months)

Using the results of phase 1, we will prepare the materials needed for phase 2, a preliminary nomenclature manual that includes the list of labels and definitions, and develop the instructions for phases 2b and 2c and training videos for phase 2c.

#### Phase 2b: decoding/interpreting behaviour change intervention protocols (four months)

This phase will generate empirical data to examine whether the list of BCTs leads to reliable identification of BCTs that can be generalised to a range of behaviours and populations.

### Research questions

1. Do researchers agree about BCTs used in published descriptions of behaviour change interventions?

2. Are these judgments reliable over time?

3. Are the proposed labels and definitions acceptable to research users?

### Participants

Participants will be 48 expert coders (half systematic reviewers, half primary researchers), each coding 20 protocols.

### Materials

We will use 40 published behaviour change intervention protocols (to allow a range of interventions sampled across health, illness, and healthcare). We will sample protocols from journals that meet the criteria of being interdisciplinary, high profile, including interventions targeting three groups (people who are healthy, ill, and health professionals), and targeting a broad range of behaviours. We propose to include protocols published between 2006 and 2008: *BMC Public Health *(51 protocols published between 2006 and 2008), *BMC Health Services Research *(33 protocols published between 2006 and 2008), *Implementation Science *(33 protocols published between 2006 and 2008), plus protocols from studies published in the *Annals of Behavioral Medicine *and the *British Medical Journal*.

### Procedure

Coders will be trained to use the nomenclature and be contacted to discuss any questions raised by it. For each of 40 interventions, each coder will indicate where a technique was used, which technique was used, and rate their confidence that it was used correctly. Coders will be asked to return all materials; one month later, they will be asked to repeat the coding task for the same intervention protocols (this will involve each expert for up to two days). There will be 12 randomly allocated pairs of coders, so that each protocol will be separately coded by 24 researchers, giving 12 sets of interrater reliability statistics for each of 40 protocols. The 480 reliability data points (12 pairs × 40 protocols) generated are sufficient to assess reliability with a 0.2 confidence interval [29].

### Analyses

For each protocol, we will establish whether the two experts agree on which BCTs have been used and whether each expert identifies the same BCTs at a second time point. Agreement will be measured by a series of kappa statistics to assess interrater reliability of technique identification at time 1 and at time 2 and within-rater test-retest reliability (http://www.psychassessment.com.au/ [accessed 5.5.08]). The sample size needed was calculated using the goodness-of-fit approach for sample size estimation [29]. For a null hypothesis of a kappa of 0.6 (*i.e.*, substantial agreement using the Landis and Koch classification [30]) versus an alternative hypothesis of kappa not equal to 0.6, a probability of rating success of 0.1, alpha = 0.05, power = 0.80, the required sample size was calculated to be 51. We will examine reliability ranges across type of coder (primary researcher, systematic reviewer) and type of protocol (target population, behaviour, length, etc.), using intraclass correlations (ICCs). No data are available to allow the calculation of an ICC for coders. However, we anticipate that the impact of clustering, if present, will be small. We will calculate an ICC on the data that we gather in order to inform the interpretation of the data.

### Acceptability/usability of the methodology

The experts will rate their experience of using the nomenclature to interpret behaviour change interventions (time taken, level of difficulty, and specific problems encountered) and will use rating scales to rate their attitude towards, confidence in, and intention to use the nomenclature. They will be asked to identify BCT definitions that remain unclear. This information will be used to clarify and refine the presentation and definitions of the nomenclature. Any ambiguities reported by the experts will inform the rewording of the technique descriptions to enhance clarity.

#### Phase 2c: encoding/writing behaviour change intervention protocols (six months)

### Research questions

1. Does using the nomenclature lead to clearer, more replicable protocols?

2. Do experts independently rate the intervention to be the same when different people write the protocol?

3. Is the nomenclature acceptable to users?

### Materials

We will use three videos showing sections of behaviour change interventions, with two people (practitioner and participant) role-playing a range of BCTs.

### Participants

Twenty-six expert intervention designers that were not involved in phase 2b will participate: 20 will write, and 6 will rate intervention protocols (estimated time per expert: one day).

### Procedures

The 20 writers will be randomly allocated to use the nomenclature (n = 10) or not (n = 10) and will each be presented with videos of three interventions incorporating a range of BCTs. They will be asked to write a description of each intervention's content in such a way that the intervention could be understood and replicated by others, resulting in 60 descriptions (20 for each intervention). The six raters will each receive a random sample of 20 of the 60 descriptions and be asked to sort them into groups that describe the same intervention using Q sort (this method enables one to detect shared ways of thinking and is especially suited where items are complex and partially overlapping) [31]. Raters will also judge each intervention description on rating scales measuring (a) ease of understanding, (b) adequacy of information required to undertake a replication, and (c) ease of identification of discrete BCTs. Acceptability will be evaluated as in phase 2b.

### Analysis

Analyses of variance will be used for each of the three rating scale outcomes to identify whether (as predicted) availability of the nomenclature leads to better-written intervention descriptions. The Q-sort data will be analysed to ascertain whether there is greater agreement about the similarity of different descriptions of the same intervention in the nomenclature group than in the nonnomenclature group. One hundred and twenty data points (20 writers × three videos × two raters) are adequate for analysis: For analysis of variance, the required number of data points for nomenclature versus no nomenclature and two replications (each protocol is judged by two raters) is 90 for a between-rater correlation = 0.4 (or N = 116 for correlation = 0.8) and power = 0.8, with alpha = 0.05 and medium effect size = 0.25. For Q-sort methods, five raters would give reliability = 0.83, assuming correlation between raters of 0.5 [32].

### Phase 3: addressing objective 3: prototype nomenclature (two months)

The outputs of phase 2 will be used to select the BCTs with demonstrable reliability (our preliminary work showed > 90% BCTs were reliable) for both identifying and reporting behaviour change interventions. We will produce a manual including the nomenclature with definitions and guidance on its use, evidence of consensus, evidence of reliability, and usability of each BCT, illustrated with examples from effective interventions. Members of the team have developed this type of manual, with different content, for researchers to use (see http://www.rebeqi.org). This previous manual was disseminated using some of the strategies described below under 'Dissemination and Implementation' and has subsequently been downloaded from the host website over 25,000 times since 1 January 2006.

### Cross-phase stream: addressing objective 4: dissemination and implementation of the prototype nomenclature (eight months over the whole three years of the study)

Our goal is to maximise awareness, understanding, and use of the nomenclature in the development, evidence synthesis, and reporting of complex interventions. This will be achieved by disseminating evidence about the benefits of the nomenclature and how to use it, promoting change in current practice, and developing the nomenclature further.

### Stakeholders

Within the first year, we will convene a meeting/teleconference with representatives from all stakeholders to optimise our implementation and dissemination strategy and build alliances with key initiatives to raise the profile of the nomenclature (*e.g.*, US Society of Behavioral Medicine's Evidence Based Medicine initiative).

### International advisory group

We will establish an international advisory group to provide input and advice at key points over the three years of the study. Members will be leading experts in researching behaviour change methods.

### Publications

Throughout the study, we will increase awareness about the nomenclature and its benefits to researchers, practitioners, policy makers, academic and professional bodies, funders, and journal editors through conference presentations, editorials, and peer-reviewed publications in academic and professional journals and *MRC Network*. We will also provide updates to stakeholders after each phase.

### Resources

We will increase understanding and use of the nomenclature among scientists, intervention designers, and practitioners by providing (a) a web-based handbook and resources for skills training (*e.g.*, video recordings of simulated interventions) and (b) training and engagement workshops providing supervised experience and recruiting through charities (*e.g.*, Cancer Research UK, British Heart Foundation), academic bodies (*e.g.*, UK Society for Behavioural Medicine), professional bodies (*e.g.*, Royal College of General Practitioners), and the National Institute for Health and Clinical Excellence's Centre for Public Health Excellence.

### Networking

We will promote action by funders and journal editors through our personal, professional and scientific networks (*e.g.*, WIDER).

### Wiki

We will promote further development of the nomenclature by upgrading and disseminating relevant evidence and asking the wider complex-interventions scientific community to provide feedback on their experiences of using it on an interactive web-based platform (Wiki) that allows ongoing iterative development by user feedback and interaction.

We have already begun working with journal editors to change editorial policy (*e.g.*, *Implementation Science *and *Addiction*) so that detailed specification of intervention content is required for publication [33-36].

We have started the development of a Wiki of BCTs to provide a web-based interactive resource to facilitate future collaboration and consensus development in refining the tools beyond the life of the proposed research. A pilot doctoral project has engaged 21 participants, providing very useful and relevant data (see interventiondesign.eu).

### Ethics and research governance

The conduct of the study will conform to relevant ethical and legal guidelines covering consent, confidentiality, and the storage of data. Ethics approval was obtained from University College London (Number: CEHP/2010A/005).

### Data preservation for sharing

All data will be preserved and its availability for use by other research teams will be publicised via the website resource and as part of our dissemination work. The data will be suitable for further analysis both in primary research and in meta-analyses. The data will be prepared to allow independent usage. We will institute an automatic registration system to track usage of the database.

## Competing interests

MPE is Co-Editor in Chief of *Implementation Science*. All decisions on this manuscript were made by another editor.

## Authors' contributions

All authors contributed to the ideas in this protocol. SM led the writing, and all authors contributed to drafts and approved the final version.
